# Is there an association between seeing incidents of alcohol or drug use in films and young Scottish adults' own alcohol or drug use? A cross sectional study

**DOI:** 10.1186/1471-2458-11-259

**Published:** 2011-04-23

**Authors:** Kate Hunt, Helen Sweeting, James Sargent, Heather Lewars, Robert Young, Patrick West

**Affiliations:** 1 MRC Social and Public Health Sciences Unit, 4 Lilybank Gardens, Glasgow, G12 8RZ, UK; 2 Cancer Risk Behaviors Group, Norris Cotton Cancer Center, Dartmouth Medical School, One Medical Center Dr, Lebanon NH 03756, USA

**Keywords:** alcohol, drugs, films, movies, adolescents

## Abstract

**Background:**

As the promotion of alcohol and tobacco to young people through direct advertising has become increasingly restricted, there has been greater interest in whether images of certain behaviours in films are associated with uptake of those behaviours in young people. Associations have been reported between exposure to smoking images in films and smoking initiation, and between exposure to film alcohol images and initiation of alcohol consumption, in younger adolescents in the USA and Germany. To date no studies have reported on film images of recreational drug use and young people's own drug use.

**Methods:**

Cross sectional multivariable logistic regression analysis of data collected at age 19 (2002-4) from a cohort of young people (502 boys, 500 girls) previously surveyed at ages 11 (in 1994-5), 13 and 15 in schools in the West of Scotland. Outcome measures at age 19 were: exceeding the 'sensible drinking' guidelines ('heavy drinkers') and binge drinking (based on alcohol consumption reported in last week), and ever use of cannabis and of 'hard' drugs. The principle predictor variables were an estimate of exposure to images of alcohol, and of drug use, in films, controlling for factors related to the uptake of substance use in young people.

**Results:**

A third of these young adults (33%) were classed as 'heavy drinkers' and half (47%) as 'binge drinkers' on the basis of their previous week's consumption. Over half (56%) reported ever use of cannabis and 13% ever use of one or more of the 'hard' drugs listed. There were linear trends in the percentage of heavy drinkers (p = .018) and binge drinkers (p = 0.012) by film alcohol exposure quartiles, and for ever use of cannabis by film drug exposure (p = .000), and for ever use of 'hard' drugs (p = .033). The odds ratios for heavy drinking (1.56, 95% CI 1.06-2.29 comparing highest with lowest quartile of film alcohol exposure) and binge drinking (1.59, 95% CI 1.10-2.30) were attenuated by adjustment for gender, social class, family background (parental structure, parental care and parental control), attitudes to risk-taking and rule-breaking, and qualifications (OR heavy drinking 1.42, 95% CI 0.95-2.13 and binge drinking 1.49, 95% CI 1.01-2.19), and further so when adjusting for friends' drinking status (when the odds ratios were no longer significant). A similar pattern was seen for ever use of cannabis and 'hard' drugs (unadjusted OR 1.80, 95% CI 1.24-2.62 and 1.57, 95% CI 0.91-2.69 respectively, 'fully' adjusted OR 1.41 (0.90-2.22 and 1.28 (0.66-2.47) respectively).

**Conclusions:**

Despite some limitations, which are discussed, these cross-sectional results add to a body of work which suggests that it is important to design good longitudinal studies which can determine whether exposure to images of potentially health-damaging behaviours lead to uptake of these behaviours during adolescence and early adulthood, and to examine factors that might mediate this relationship.

## Introduction

In high income countries there is concern about the consequences of excessive alcohol consumption [1], especially in youth when these behaviours are common [1-4] and may track into adulthood [5,6]. There is evidence of a "dramatic rise" in alcohol consumption in young people in the west of Scotland and in the UK more broadly [7]. The reduction of alcohol (mis)use, and binge drinking in particular, are priorities for the British Government [3]. This reflects concerns about public drunkenness and anti-social behaviour on the one hand, and the longer term health effects of excessive drinking, such as increased mortality in heavy drinkers [8].

There is also evidence from Scotland of an increase in the lifetime prevalence (ever use) of illicit drugs in recent decades, with ever-use of cannabis by young adulthood being much more common than ever-use of other drugs [9]. Cannabis use in young people is associated with psychotic symptoms and dependence on other illicit drugs [10,11], although there is debate over its health consequences [12]. Among young adults who have been long-term drug users, there is evidence of poor self-rated health and increased mortality [13,14].

This evidence on increasing substance use in adolescents and young adults, together with the lack of effective treatment for substance dependence [15], raises questions about which factors facilitate the uptake of excessive alcohol and drug use.

Media portrayals are one potential influence shaping young people's views of various behaviours. However, it has been demonstrated that portrayals of substance use in films are often unrealistic, as has been well documented for smoking [16-18]. They often glamourise smoking and make smoking appear to be more prevalent than contemporary figures support. Thus, despite the dramatic fall in adult smoking in the UK and USA since the 1950s, it has been suggested that smoking in films was as common in 2002 as in 1950 [19]. Smoking imagery declined in top US box office hits between 1996 and 2004, but not within films intended for youth audiences [20]. Similar findings have been reported for the most popular films in the UK, showing that despite a substantial fall between 1989 and 2008 overall, tobacco imagery appeared in 70% of all films, and predominantly in films categorised as suitable for children and young people [21]. This has alerted health professionals and policy-makers to the potential of media images to shape substance use in young people [22]. Evidence is now building to suggest a causal link between viewing images of smoking in films and young people's initiation of smoking [22-26]. To date, little attention has been paid to the influence of film images of other behaviours, such as alcohol and illicit drug use, on young people's own use of these substances.

Alcohol consumption is also very commonly portrayed in films, including in (US) G-rated (General Audience) [27] and animated [28] films. A content analysis of 100 of the top grossing US films between 1986 and 1994 reported that 96% had references that supported alcohol use, and 79% included at least one character who used alcohol. Whilst incidents of alcohol use were common, portrayals of the hazards of drinking were not reflected [29]. Similarly, a study of the most popular US film rentals from 1996-7 found 93% included alcohol use and 22% illicit drug use; in 12% of films one or more of the major characters used drugs and 65% of adult characters used alcohol; and in 43% of films alcohol use was portrayed as a positive experience [30]. A content analysis of the top grossing US films from 1999-2001 found 15% of teen characters used illicit drugs and again were unlikely to be shown as suffering any consequences (positive or negative, short or long-term) of their drug use [31].

In very recent years a few studies have reported an association between exposure to alcohol images in films and young people's own alcohol consumption [32-35]. These studies followed earlier ones which had demonstrated an effect of exposure to alcohol advertising, marketing and portrayals on young people's subsequent drinking behaviours [36]. Thus, in the USA, a strong relationship was seen between film alcohol exposure and onset of drinking in 3577 10-14 year olds who were never drinkers at baseline [32]. Cross-sectional associations between film alcohol exposure and drinking were observed in 5581 13-year olds from 27 schools in Germany. After adjustment (for socio-demographic, parenting and personal characteristics and friends' drinking), the odds ratios were 1.47 (95% confidence interval [CI] 1.19-1.82), 2.12 (95% CI 1.75-2.57) and 2.95 (2.35-3.70) for drinking without parental knowledge (comparing the higher three quartiles of exposure to the lowest) and 1.42 (0.93-2.28), 1.84 (1.27-2.67) and 2.59 (1.70-3.95) for binge drinking [33]. To our knowledge, no studies have reported on exposure to images of illicit drug use and own drug use.

Here we report a cross-sectional analysis which investigates the association between exposure to images of a) alcohol and b) drugs in films and a) current drinking and b) ever use of drugs in young adults (aged 19) living in the UK. We have previously reported a lack of association between exposure to smoking in films and smoking in these young adults [37]. As a number of factors may confound any relationship between film exposure and substance use [36], we adjust for gender, background characteristics, personal characteristics, friends' substance use and time spent watching television, videos or dvds.

## Methods

### Sample

Data are from the *West of Scotland 11 to 16/16+ Study*, a longitudinal study of health and lifestyles in a single year cohort [38]. Respondents were recruited in 1994-5 during their final year of primary schooling (age 11) and re-surveyed at ages 13 and 15 (in the 43 secondary schools to which they transferred), and at age 19 after leaving school. At 11, parental questionnaires were completed for 86% of the sample. The study received approval from the University of Glasgow Ethics Committee for Non-clinical Research Involving Human Subjects and (for school-based stages) participating Education Authorities and schools. Respondents were invited to take part via letters with information sheets detailing the survey procedures. Prior to participation they signed a consent form confirming that they had read the information sheet, had the study explained to them and understood what it involved, that the information they would provide was confidential and would be identifiable only by an ID number and that they could choose not to answer any questions they wished.

Because of the school-based nature of the sample, the sampling scheme involved several elements to ensure representativeness at both the primary and secondary school stages, as reported elsewhere [7]. In brief, the survey used a reverse-sampling procedure which randomly selected the 43 secondary schools stratified by level of deprivation and religious denomination, with a separate stratum for independent and state-run schools. These schools were used to select a random sample of 'feeder' primary schools (traditionally linked with the secondary schools), together with primaries making a high number of parental placing requests. Within these 135 primary schools, classes were randomly selected, with all pupils in selected classes eligible to participate. Of the 2793 pupils who attended the targeted secondary schools, 2586 (93%) participated in the baseline (age 11) survey, 85% in the survey at age 13, and 79% in the survey at age 15. As expected, losses to follow-up increased in the post-school period, reducing the sample size to 1256 (45%) at age 19. Full details of the sampling strategy are available elsewhere [39].

The baseline sample was representative of 11 year olds in the study area in respect of sex and socio-economic status (SES) [40]. Differential attrition made subsequent waves less representative; for example, attrition was higher among lower SES groups, school truants, early school leavers, and smokers. Probabilistic weights have been derived at each wave to compensate for non-response [40,41], adopting the system of weighting proposed by Little and David [42]. As these factors could be related to alcohol and drug use, we report results based on weighted data at age 19 (n = 1006 - because only those who completed all waves were assigned a weight). Unweighted analyses are available on request.

Each school-based survey included self-completion questionnaires administered in exam-type conditions. At age 19, respondents were interviewed by nurses using computer aided personal interviews.

### Measures

#### Exposure to alcohol and drug-taking in films

To estimate the amount of alcohol and illicit drug use that the respondents had seen in films ('film alcohol exposure' and 'film drug exposure') we aimed to replicate methods developed by Sargent and colleagues [23-25,43] as closely as possible. At age 19, respondents were asked to indicate in a self-completion questionnaire whether they had seen each of a unique list of 50 films randomly selected from a sample of 601 films released between 1988 and 1999; hence each respondent's list of 50 was different. The 601 films included the USA's 25 top box-office hits from 1988 to 1995 (n = 200); the top 100 box-office hits in 1996, 1997 and 1998 (n = 300); the top 50 box-office hits from the first half of 1999; and 51 additional films which featured stars popular amongst adolescents [25].

Trained coders have recorded the number of seconds of alcohol and drug use in each film as described elsewhere [32]. Alcohol use was defined as consumption of a beverage that was clearly alcoholic, implied possession of such a beverage (e.g. a character sitting in a bar with a filled beer glass), or purchasing alcohol. Excluded were occasions when a character had an empty alcoholic beverage container (e.g. empty beer bottle) or when alcoholic beverage containers were displayed but were not implied as being consumed (e.g. bottles shown above a bar). Drug use included actual or implied use (e.g. a character saying that they had used drugs just prior to a scene) or specific preparation for use (e.g. rolling a joint) as well as drug dealing. It also included use of drugs prescribed for another person, but not use or misuse of a person's own prescription drug.

An index of film alcohol use was calculated by summing the seconds of alcohol use in the films that each respondent had seen from his/her list of 50 films. This number was divided by the seconds of alcohol use they would have viewed if they had seen all 50 films on their list. This proportion was multiplied by the seconds of alcohol use in the full sample of 601 films, to provide an estimated exposure to alcohol in all 601 films given their viewing habits (see [32]). A separate index of film drug use was calculated in an analogous fashion (i.e. summing the seconds of drug use in each film and dividing it by the number of seconds of drug use they would have viewed if they had seen all 50 films on their list). The total estimated exposures for each respondent were translated into minutes. One case who did not complete a film list and two who reported having seen all 50 films on their list were excluded (resulting weighted N = 1002). The estimated film exposure variables were then classified into quartiles. Cut offs for the film drinking exposure were: 0.4-476 minutes for the lowest, 477-691 for the 2^nd^, 692-931 for the 3^rd^, and 931-2017 for the highest quartile; those for the film drugs exposure were 0-7 minutes, 8-30 minutes, 30-70 minutes and 70-175 minutes.

#### Alcohol

At age 19, current drinkers reported the quantity of a range of alcoholic drinks consumed each day over the past week. This was summed over the last week; never and ex-drinkers were assumed to have consumed 0 units. Dichotomous measures were derived. We followed the UK Royal College of Psychiatrists' guidelines to define **'binge drinking' **(females were defined as a binge drinker if they had consumed over 6 units in any single day in the last week, and males if they had consumed over 9 units [1,44]). **'Heavy weekly drinkers' **were those exceeding current guidelines (over 14 units per week for females, over 21 units for males) [1,44].

#### Drug use

Respondents indicated which drugs they had ever used from a list which included common street names (e.g. cannabis [hash, grass, dope]; temazepam [jellies, ruggers, eggs, Gellphix]). Because of the differing social characteristics of people who have only ever used cannabis vs other drugs [9], we report here two separate outcomes: **ever use of cannabis **and **ever use of 'hard' drugs**, defined, following recommendation by the Prevention Working Group of the UK Advisory Council on the Misuse of Drugs [9] as temazepam, tranquillisers, heroin, methadone, temgesic, cocaine, crack and morphine or opium.

#### Parental social class

Occupational data from parents at age 11 were used to derive a head of household classification (using father's current occupation or previous if not currently working, or if no father, the mother's current or previous occupation) (from herein referred to as 'social class'). Where no parental data were available, information from the young person (at age 11) on current parental occupation was utilised; the reliability of these data is high [45]. Social class data (classified using the UK Registrar General's Classification of Occupations [46]) were collapsed into four categories: non-manual (white collar and professional) occupations (class I, II and IIINM); skilled manual (blue collar) (class IIIM); semi-skilled and unskilled manual (class IV and V); and missing.

#### Parental structure

At age 15 respondents reported which parental figure(s) they lived with (classified here as: both birth parents; one birth parent and new partner; one birth parent alone (the majority) or other relatives (e.g. a grandparent)). The very few cases with no parent (e.g. with foster parents) were excluded because information on parental care/control and household variables could not be consistently evaluated by the respondents.

#### Parental Bonding Inventory

At 15, respondents completed the Brief Parental Bonding Instrument (PBI) [47] which provides scores for parental care and (over)control ranging from 0-8 (higher scores representing greater perceived care and control). Each scale was collapsed into three categories for crosstabulations but used as a continuous variable in the logistic regressions.

#### Attitudes to risk and rule-breaking

At 15, respondents rated themselves in relation to risk-taking ('I take risks') and rule-breaking ('I am a rule breaker'), with response categories 'very true', 'true', 'untrue' and 'very untrue'.

#### Qualifications by age 19

Respondents were dichotomised into those who had obtained any 'Highers' at school (Scottish qualifications, generally taken at age 16-17, required for entry into higher education) vs none.

#### Friends' alcohol and drug use

At 19, respondents reported how many of their friends engaged in various activities, with seven categories ranging from 'none' to 'all'. Two dichotomous measures were derived for the crosstabulations: whether half or more of their friends drank, and used cannabis.

#### TV, video and dvd use

At 19, respondents reported how many hours each week and weekend day they usually spent watching television, videos or dvds. The total hours per week were categorised as 0-9, 10-19, 20-29, 30-39 and 40+ hours for crosstabulations.

### Analysis

Crosstabulations were used to compare the proportions for the four outcomes at age 19 according to quartiles of alcohol and drug exposure (as appropriate), and potential confounders. A series of logistic regression models was then run for each outcome. Multivariate models were built sequentially. First, the unadjusted relationship with the relevant film exposure was assessed. Subsequent models adjusted for gender, then additionally for: background variables (social class, parental structure, care and control); personal characteristics (risk-taking, rule-breaking, qualifications); friend's drinking or drug use; and finally for hours per week watching television, videos or dvds. We present weighted data but analyses using unweighted data produced similar results (available on request). Crosstabulations and logistic regression analyses excluded respondents who had missing data on any of the potential confounders in the final multivariate model (resulting N in respect of heavy drinking = 922; binge drinking = 928, ever use of cannabis and ever use of 'hard' drugs = 926).

## Results

Basic descriptive characteristics of the sample are shown in Table 1. Substance use was common. A third (33%) of the young adults were classed as 'heavy drinkers' and half (47%) as 'binge drinkers'. Over half (56%) reported ever use of cannabis, but many fewer (13%) reported ever use of one or more of the 'hard' drugs listed. Almost all (93%) reported that half or more of their friends drank alcohol (equivalent figure for ever use of cannabis, 21%).

**Table 1:** Descriptive data: frequency of last week heavy and binge drinking, ever cannabis and 'hard' drugs, and of potential confounders.

		N	(%)
**Outcome variables**			
			
Heavy drinking previous week (age 19) ^a^	Yes	326	(32.9)
	No	663	(67.1)
			
Binge drinking previous week (age 19) ^b^	Yes	466	(46.7)
	No	532	(53.3)
			
Ever use of cannabis (age 19)	Ever	564	(56.3)
	Never	438	(43.7)
			
Ever use of 'hard' drug use (age 19)	Ever	131	(13.0)
	Never	871	(87.0)
			
**Potential confounders **			
			
Gender	Male	502	(50.1)
	Female	500	(49.9)
			
Parental social class (age 11)	Non-manual	413	(41.2)
	Skilled manual	299	(29.9)
	Semi/unskilled manual	225	(22.4)
	Missing	65	(6.5)
			
Parental structure (age 15)	Both birth parents	696	(70.6)
	Birth mother/father and new partner	115	(11.7)
	Birth mother/father alone or with other relatives	174	(17.7)
			
Parental care (age 15)	Low	254	(25.4)
	Medium	426	(42.6)
	High	319	(32.0)
			
Parental control (age 15)	Low	364	(36.7)
	Medium	256	(25.8)
	High	373	(37.5)
			
'I take risks' (age 15)	Very untrue	50	(5.0)
	Untrue	304	(30.5)
	True	539	(54.0)
	Very true	105	(10.5)
			
'I am a rule-breaker'	Very untrue	250	(25.1)
	Untrue	487	(49.1)
	True	215	(21.6)
	Very true	41	(4.2)
			
'Highers' (age 19)	None	434	(43.3)
	Any	567	(56.7)
			
Friends' drinking status (age 19)	None - a few	67	(6.8)
	Half or more	923	(93.2)
			
Friends' use of cannabis (age 19)	None - a few	777	(78.9)
	Half or more	208	(21.1)
			
Hours per week tv, videos, dvds (age 19)	0-9	166	(16.8)
	10-19	349	(35.5)
	20-29	277	(28.1)
	30-39	98	(10.0)
	40 or more	94	(9.5)

^a ^Males drinking over 21 units, females 14 in the past week (WHO guidelines)

^b ^Males drinking 10+ units, females 7+ on any one day in the past week

Respondents had seen a mean of 19.0 (SD = 7.3, range 1-44) of the 50 films presented to them; mean film alcohol and drug exposures were 726 minutes (12.1 hours) and 45 minutes respectively. The mean number of films was higher for males (20.8) than females (17.3, F = 60.5, p = .000) and males' film alcohol and drug exposures were higher (770 vs. 682 minutes, F = 16.5, p = .000 and 51 vs. 38 minutes, F = 23.1, p = .000 respectively). There were no social class differences for films seen or film alcohol exposure, but film drug exposure was higher in those from higher social class backgrounds (non-manual = 51, skilled manual = 40, semi/unskilled manual = 41 minutes, F = 6.6, p = .001). There was a positive correlation between the film alcohol and drug exposure measures (r = .510).

Table 2 reports the percentage of heavy and binge drinkers by quartile of film alcohol exposure, and the percentage of ever users of cannabis and ever users of 'hard' drugs by film drug exposure. The p values reported in the table relate to heterogeneity within the groups, but we also tested for linear trends. In the cross-tabulations, the tests for linear trends in the percentage of heavy drinkers (p = .018) and binge drinkers (p = 0.012) by film alcohol exposure quartiles (see Table 2) were statistically significant. Similarly, there was an increase in the percent who had ever used cannabis with each quartile of film drugs exposure (linear trend p = .000). The percentage who had used 'hard' drugs was also highest in the highest quartile of film drug exposure (16%), but with less evidence of a stepwise increase (linear trend p = .033).

**Table 2:** Alcohol and drug use by predictor variables - percentages (significance of chi-square).

	Heavy drinking (last week)		Binge Drinking (last week)		Cannabis Use (ever)		'Hard' drug use (ever)	
	%	*(sig)*	%	*(sig)*	%	*(sig)*	%	*(sig)*
**Alcohol use in movies seen**								
Quartile 1	29.9		41.4					
Quartile 2	29.8		44.5					
Quartile 3	31.7		46.3					
Quartile 4	40.2	*(.054)*	53.0	*(.079)*				
**Drug use in movies seen**								
Quartile 1					49.8		11.0	
Quartile 2					47.8		8.2	
Quartile 3					59.3		13.9	
Quartile 4					64.2	*(.001)*	16.0	*(.059)*
**Gender**								
Male	40.2		52.7		63.4		16.0	
Female	25.7	*(.000)*	40.1	*(.000)*	47.3	*(.000)*	8.5	*(.000)*
**Parental social class (age 11)**								
Non-manual	32.2		43.4		53.1		9.2	
Skilled manual	34.2		48.4		57.4		14.1	
Semi/unskilled manual	31.3		47.0		58.2		16.5	
Missing	37.3	*(.809)*	53.8	*(.387)*	48.0	*(.382)*	8.2	*(.037)*
**Parental structure (age 15)**								
Both birth parents	32.7		45.5		54.3		12.1	
Birth mother/father and new partner	40.2		54.9		62.7		12.7	
Birth mother/father alone or with other relatives	28.7	*(.154)*	44.1	*(.172)*	54.4	*(.270)*	12.1	*(.984)*
**Parental Bonding Inventory - care (age 15)**								
Low	35.0		49.5		62.2		18.0	
Medium	33.8		47.9		56.8		10.2	
High	30.0	*(.418)*	41.5	*(.128)*	47.8	*(.003)*	10.7	*(.012)*
**Parental Bonding Inventory - control (age 15)**								
Low	34.5		49.1		54.1		13.6	
Medium	30.1		43.3		53.8		10.4	
High	33.1	*(.537)*	45.5	*(.365)*	57.3	*(.605)*	12.1	*(.502)*
**'I take risks' (age 15)**								
Very untrue	13.0		21.7		40.4		6.5	
Untrue	22.8		37.2		39.2		5.7	
True	37.3		51.8		64.0		12.9	
Very true	49.5	*(.000)*	55.4	*(.000)*	64.4	*(.000)*	29.7	*(.000)*
**'I am a rule breaker' (age 15)**								
Very untrue	22.4		34.2		37.0		6.8	
Untrue	31.0		46.2		53.6		7.7	
True	47.9		61.3		74.2		23.2	
Very true	42.5	*(.000)*	47.5	*(.000)*	87.5	*(.000)*	41.0	*(.000)*
**'Highers' (age 19)**								
None	38.2		49.9		64.3		21.1	
Any	29.2	*(.004)*	43.7	*(.065)*	48.8	*(.000)*	5.9	*(.000)*
**Friends' drinking status (age 19)**								
None - a few	4.8		6.3					
Half or more	34.9	*(.000)*	49.2	*(.000)*				
**Friends' cannabis use (age 19)**								
None - a few					47.6		7.1	
Half or more					85.2	*(.000)*	32.3	*(.000)*
**Hours per week tv, videos, dvds (age 19)**								
0-9	31.2		43.9		53.5		7.6	
10-19	33.4		48.6		57.3		12.1	
20-29	31.3		42.6		53.8		14.4	
30-39	32.6		46.8		58.9		17.9	
40 or more	39.8	*(.678)*	51.8	*(.475)*	50.6	*(.702)*	8.6	*(.089)*
***(N)***	***(922)***		***(928)***		***(926)***		***(926)***	

(Analyses restricted to those with valid responses on all variables relevant to each outcome.)

Male gender and perceiving oneself as a risk-taker and rule-breaker were associated with all four substance use measures (p < 0.001 in all cases), and having no 'Highers' at 19 with all (p = 0.004 for heavy drinking, and p < 0.001 for ever use of cannabis, and ever use of hard drugs) except binge drinking (p = 0.65). Respondents from manual class backgrounds were more likely to have used 'hard' drugs (p = 0.037), and those reporting lower parental care were more likely to have ever used both cannabis (p = 0.003) and 'hard' drugs (p = 0.012). Friends' drinking and cannabis use were strongly associated with own drinking and drug status respectively. There were no associations between the substance use measures and parental structure, parental control, or hours per week watching television, videos or dvds.

Table 3 shows the results of the logistic regression models for each outcome, both before and after adjusting for potential confounding or mediating variables. We consider the alcohol outcomes first. In the unadjusted model, those in the highest quartile of film alcohol exposure were more likely to be classed as both heavy and binge drinkers ((OR = 1.56, 95% CI 1.06-2.29) and 1.59 (1.10-2.30) respectively, compared with the lowest quartile) on the basis of their reported alcohol consumption the previous week. Adjustment for gender reduced the associations, but further adjustment for background characteristics returned the odds ratios for the drinking measures to the unadjusted levels. Adjusting for risk-taking, rule-breaking and qualifications, and particularly for friends' drinking status, reduced the odds ratios, but further adjustment for hours watching television, videos or dvds made no difference to the associations. In this final model only gender (OR for females 0.59 (95% CI 0.43-0.80) for heavy drinking and 0.64 (95% CI 0.48-0.85) for binge drinking) and friends' drinking status (OR for half or more friends drinking 1.44 (95% CI 1.27-1.64) for heavy drinking and 1.54 (95% CI 1.36-1.73) for binge drinking) had odds ratios which did not include unity (1.00), i.e. film alcohol exposure was no longer significantly associated with heavy or binge drinking. (Full tables showing OR and 95% CI for all variables included in all models available on request.)

**Table 3:** Results of logistic regression models including film exposure quartiles, (a) unadjusted and (b-f) adjusted for potential confounders - ORs (95% CIs).

	Heavy drinking (last week)		Binge drinking (last week)		Cannabis use (ever)		'Hard' drug use (ever)	
	OR	(95% CIs)	OR	(95% CIs)	OR	(95% CIs)	OR	(95% CIs)
**Alcohol/drug use in movies seen**								
								
**(a) unadjusted**								
Quartile 1	1.00		1.00		1.00		1.00	
Quartile 2	0.99	(0.67-1.47)	1.12	(0.78-1.62)	0.92	(0.64-1.32)	0.73	(0.39-1.36)
Quartile 3	1.08	(0.73-1.61)	1.21	(0.84-1.75)	1.46	(1.01-2.10)	1.30	(0.75-2.26)
Quartile 4	1.56	(1.06-2.29)	1.59	(1.10-2.30)	1.80	(1.24-2.62)	1.57	(0.91-2.69)
								
**(b) adjusted for gender**								
Quartile 1	1.00		1.00		1.00		1.00	
Quartile 2	1.00	(0.67-1.50)	1.14	(0.79-1.64)	0.92	(0.64-1.33)	0.73	(0.39-1.40)
Quartile 3	1.07	(0.71-1.59)	1.20	(0.83-1.74)	1.38	(0.95-1.99)	1.21	(0.69-2.12)
Quartile 4	1.47	(0.99-2.17)	1.51	(1.04-2.19)	1.66	(1.14-2.43)	1.42	(0.82-2.46)
								
**(c) adjusted for gender and background** **(parental social class, parental** **structure, PBI care and control)**								
Quartile 1	1.00		1.00		1.00		1.00	
Quartile 2	1.05	(0.70-1.58)	1.20	(0.83-1.75)	0.92	(0.64-1.34)	0.71	(0.38-1.34)
Quartile 3	1.12	(0.74-1.68)	1.26	(0.86-1.84)	1.39	(0.95-2.02)	1.24	(0.70-2.18)
Quartile 4	1.55	(1.04-2.30)	1.59	(1.09-2.33)	1.67	(1.14-2.46)	1.50	(0.86-2.62)
								
**(d) adjusted for gender, background** **and personal characteristics** **(take risks, rule-breaker, 'Highers')**								
Quartile 1	1.00		1.00		1.00		1.00	
Quartile 2	0.93	(0.61-1.41)	1.11	(0.76-1.63)	0.78	(0.52-1.15)	0.61	(0.31-1.19)
Quartile 3	1.04	(0.68-1.57)	1.19	(0.81-1.74)	1.27	(0.86-1.89)	1.16	(0.63-2.13)
Quartile 4	1.42	(0.95-2.13)	1.49	(1.01-2.19)	1.49	(0.99-2.24)	1.40	(0.76-2.56)
								
**(e) adjusted for gender, background,** **personal characteristics and friends'** **drinking status/cannabis use (as appropriate)**								
Quartile 1	1.00		1.00		1.00		1.00	
Quartile 2	0.80	(0.52-1.23)	0.94	(0.63-1.41)	0.74	(0.48-1.13)	0.54	(0.26-1.12)
Quartile 3	0.94	(0.61-1.44)	1.06	(0.71-1.58)	1.21	(0.78-1.86)	1.03	(0.53-1.99)
Quartile 4	1.25	(0.82-1.89)	1.28	(0.86-1.91)	1.34	(0.85-2.09)	1.24	(0.64-2.39)
								
**(f) adjusted for gender, background,** **personal characteristics, friends'** **drinking/cannabis and own tv, dvd** **or video hours**.								
Quartile 1	1.00		1.00		1.00		1.00	
Quartile 2	0.80	(0.52-1.23)	0.95	(0.63-1.41)	0.76	(0.49-1.17)	0.55	(0.26-1.14)
Quartile 3	0.94	(0.61-1.45)	1.07	(0.71-1.60)	1.26	(0.82-1.96)	1.07	(0.55-2.09)
Quartile 4	1.25	(0.82-1.90)	1.29	(0.86-1.92)	1.41	(0.90-2.22)	1.28	(0.66-2.47)
***(N in each analysis)***	***(922)***		***(928)***		***(926)***		***(926)***	

We turn now to consider the relationship between exposure to film images of illicit drug use and ever-use of cannabis and 'hard' drugs. In the unadjusted model, ever use of cannabis showed a stepped association with film drug exposure (OR for third and highest, compared with the lowest quartile of film drug exposure = 1.46 (95% CI 1.01-2.10) and 1.80 (95% CI 1.24-2.62)). Adjustment for gender attenuated the association, whereas adjusting additionally for family background made little difference. The OR was further attenuated (with 95% confidence which included unity) after adjusting for personal characteristics, friends' reported cannabis use, and then tv/dvd/video watching (see table 3). In the final model having any 'Highers' (OR 0.56, 95% CI 0.39-0.80), seeing oneself as a rule-breaker (OR 10.70, 95% CI 3.43-3.38, comparing those saying 'very true' as compared with those saying 'very untrue'), and reporting that half or more of one's friends used cannabis (OR 2.14, 95% CI 1.87-2.45) were the only ORs in the model with 95% confidence intervals that did not include unity.

For 'hard' drug use the confidence intervals for the unadjusted ORs in third (OR 1.30, 95% CI 0.75-2.26) and highest (OR 1.57, 95% CI 0.91-2.69) quartiles overlapped with unity. Although the odds were greatest in the highest film drug exposure quartile in each of the models for ever use of 'hard' drugs, none of the associations reached conventional levels of significance, even in the unadjusted model. In the final model hard drug use was significantly inversely associated with having any 'Highers' (OR 0.26, 95% CI 0.15-0.45), and positively associated with seeing oneself as a rule-breaker (OR 3.02, 95% CI 1.05-8.69, comparing those saying 'very true' as compared with those saying 'very untrue') and reporting that half or more of one's friends used cannabis (OR 2.10, 95% CI 1.76-2.51).

## Discussion

In this cross-sectional analysis, we have demonstrated an association between film exposure to alcohol and both binge and heavy drinking in young adults, and, to our knowledge for the first time, an association between film exposure to illicit drugs and ever use of cannabis. These associations persisted after adjusting for gender, social class, family structure and levels of parental control, but not after adjusting for other variables, including personal characteristics such as risk-taking, rule-breaking and achievement of school qualifications, and in particular friends' substance use. It is somewhat difficult to know how to interpret these attenuations in the associations, particularly in this cross-sectional analysis. It is likely, for example, that young people who drink heavily or take drugs are not only more inclined to do this in the company of like-minded friends, but they may also share, or develop similar tastes in cultural representations of substance use with them, which may in turn determine the kinds of films they choose to watch. On the other hand, portrayals of substance use could directly influence an individual's uptake of drinking and drug use which could itself influence the friendship groups that they choose to maintain or develop.

The cross-sectional nature of the analysis thus means that it is not possible to establish the direction of causality. Even before concerning ourselves with the impact of potential mediating or confounding factors, we cannot distinguish here between two plausible but competing explanations, either that film images of substance use may influence behaviours or that people who have already adopted particular patterns of substance use may choose to watch films that reflect similar lifestyles and values. Furthermore, it is important to acknowledge that images of substance use in films occur within a wider media context in which a vast array of different images are portrayed over time from a variety of sources (including magazines, TV, newsprint, websites and social messaging sites). A few other studies (e.g. [32,33]), one including prospective data [32], have reported an association between film alcohol exposure and drinking in younger adolescents, using similar methods. A German study (mean age 13) obtained much stronger associations between film alcohol exposure and measures of drinking, before and after adjustment for a comparable set of potential confounders [33].

Our findings of some association between exposure to film images of alcohol and illicit drugs and young people's own substance use in this cross-sectional analysis are of interest, particularly because, in contrast to other studies which have reported to date (e.g. [23-26]), we did not see any association in this study population between exposure to smoking in films and young people's own smoking at age 19 [37]. We speculated that this lack of association with smoking may be attributable to several factors; these factors could also explain the smaller association we observe in this UK study between film alcohol exposure and drinking in comparison with the USA and Germany.

First, there are methodological issues, one of which relates to respondent age. Our study differs from previous research studies which have focussed on (early) adolescent experimentation with smoking and drinking. It is plausible that, by age 19, other influences (e.g. direct observation or substance use amongst peers) could have had such a strong effect that the impact of exposure to these behaviours in films is 'swamped'. Young adults may also have a more sophisticated and critical reading of media images which makes them more resistant to their effects. A second methodological issue relates to the timing of the film exposure. We used coding of substance use in films completed by our American colleagues at the time of our fieldwork (2002-4). At this point coding was only available on films up to and including 1999 (when our sample were aged 15). Hence we missed exposures to more contemporaneously released films.

Our second group of potential explanations for a lack of an association between smoking in films and own smoking in these young people [37] related to the cultural environment and the prevalence and social prominence of the behaviours in question. Although the mass film industry is increasingly globalised, it is plausible that Scottish viewers empathise less with Hollywood film stars, or are distanced from American culture. Fictional or real-life visual portrayals of substance use in TV programmes (such as soap operas), popular with young people in the UK, may be more salient in the Scottish context.

Another potential difference lies in the prevalence of substance use in the various countries which have been studied. Scotland is commonly described as having an 'alcohol culture'. Compared with most other European countries, where levels have remained static or fallen over the last 10-15 years, alcohol consumption has increased rapidly in the UK, and within the UK, rates are highest in Scotland [Scottish Government Health & Community Care website; http://www.scotland.gov.uk/Topics/Health/health/Alcohol]. Similarly, rates of drug use in the UK are higher than most other European countries [European Monitoring Centre for Drugs and Drug Addiction, Statistical Bulletin 20008; http://www.emcdda.europa.eu/stats08]. This widespread recreational drug use, regardless of social background has been described as 'normalisation' [48]. Against such a background, any impact of the portrayal of substance use in films may be diminished.

Additional caveats that we raised in our previous paper on smoking [37] are also relevant here: we have no measure of how accurate young adults' recall of the films they had seen was; and we did not record whether films had been viewed once or repeatedly. Also alcohol and drug use were self-reported in the study (as in most similar studies), although our interviewers went to some lengths to ensure confidentiality and privacy whilst reporting on substance use. Furthermore, alcohol use measures were based on reports of consumption in the last week and this may not have been representative of the usual pattern and frequency of drinking in every individual.

Other limitations that we have raised earlier in this paper are important to rehearse. There was considerable and differential attrition between the first wave of the study (when 11 year old pupils were representative of all 11 year olds in the areas in which they lived) and the wave of data collection at age 19 years. Although we selected a weighting system designed to address differential attrition, it is possible that some residual attrition bias remains. For the alcohol variables we were able to use current measures of consumption as our outcome, whilst for the drug use variables we were only able to analyse ever-use. In the latter case we cannot know when this drug use took place or for how long it was a feature of the young person's life.

Our measures of film exposure are comparable to those reported previously. For example, a study of American 10-14 year olds, based on the same parent film sample reported that respondents had seen a median of 16 of the 50 films on their unique list (compared with 19 in our study), which translated into a median exposure to alcohol use of 8.3 hours in the sample of 601 films (compared with a mean exposure of 12.1 hours in our study). The relatively higher alcohol exposure would be expected, given the nature of films likely to have been watched by the older adolescents in our study.

## Conclusion

Our finding of an association between estimated exposure to film images of alcohol use and young people's current use of alcohol from this cross-sectional study is consistent with findings from other recent studies. The association we report for exposure to film images of illicit drugs and ever use of cannabis suggests that this may be an important relationship to explore in future well-designed longitudinal studies which are able to examine whether exposure to images of drugs in films is related to the initiation of illicit drugs use. Such studies could also explore whether the types of images (e.g. 'glamourised' or 'normalised' images of alcohol and illicit drug use as compared with negative or neutral images) affect different groups of young people in different ways.

## Pre-publication history

The pre-publication history for this paper can be accessed here:

http://www.biomedcentral.com/1471-2458/11/259/prepub
